# Performance Metrics of Anaerobic Power in Professional Mixed Martial Arts (MMA) Fighters

**DOI:** 10.3390/jfmk10030358

**Published:** 2025-09-18

**Authors:** Jessica Hanflink, Corey A. Peacock, Gabriel J. Sanders, Jose Antonio

**Affiliations:** 1 Dr. Kiran C. Patel College of Osteopathic Medicine, Nova Southeastern University, Fort Lauderdale, FL 33328, USA; jh3610@mynsu.nova.edu (J.H.); cpeacock@nova.edu (C.A.P.); 2 Exercise Science, University of Cincinnati, Cincinnati, OH 2600, USA; sandersgj@ucmail.uc.edu

**Keywords:** combat sports, exercise physiology, athlete monitoring, countermovement jump, explosive power

## Abstract

**Background:** Mixed martial arts (MMA) requires athletes to generate repeated bursts of high-intensity effort with minimal recovery time. Despite the sport’s reliance on anaerobic power, there are minimal data assessing pre-competition physiological capacity in MMA fighters. This study aimed to evaluate anaerobic performance using the Wingate Anaerobic Test (WAnT) and Countermovement Jump (CMJ) in professional MMA athletes, and to examine relationships between performance metrics across weight classes. **Methods:** Twelve professional male MMA fighters (age 29.00 ± 4.80 years, weight 85.60 ± 13.90 kg) completed both CMJ and WAnT assessments using sensor-integrated devices (Just Jump mat and Wattbike Pro). CMJ height and WAnT variables (peak power, average power, and fatigue index) were measured. Pearson correlations were used to examine the relationships between CMJ and Wingate outputs. Independent *t*-tests compared performance between lighter (<83.9 kg) and heavier (≥83.9 kg) weight groups. **Results:** CMJ performance showed significant positive correlations with both average power (r = 0.71, *p* < 0.001) and peak power (r = 0.61, *p* = 0.004). Peak power was also positively correlated with fatigue index (r = 0.84, *p* < 0.001), suggesting greater fatigue in higher power-producing athletes. Finally, the heavier weight group of fighters produced significantly (*p* = 0.03) more peak power when compared to the lighter weight group. **Conclusions:** The findings support the use of CMJ and WAnT testing as practical tools for evaluating anaerobic performance in MMA athletes. These assessments can help guide individualized training strategies, particularly when accounting for weight group specific differences in power and fatigue dynamics.

## 1. Introduction

MMA is a complex sport encompassing a unique combination of striking and grappling; the fighters must engage in an equally unique combination of physiological training to develop their fighting skills [1,2,3,4,5]. There is a wide range of competitive styles within the same sport; as such, a single test would not be sufficient to assess the physiologic capacity of an individual [6]. While VO_2_ max is commonly used to assess cardiovascular fitness, some researchers suggest that anaerobic thresholds, including ventilation thresholds, may be more relevant indicators of MMA performance. However, this claim has yet to be thoroughly validated between research studies [7]. MMA fights range from 15–25 min, with championship bouts consisting of five 5 min rounds and non-championship bouts featuring three 5 min rounds. Despite reliance on aerobic metabolism with increasing rounds, there is a strong predominance for anaerobic metabolism in high intensity efforts, with final efforts of repeat sprint protocols still relying on anaerobic energy [8]. Because MMA fighters repeatedly transition between maximal exertion and short recovery periods, their ability to sustain performance depends not only on anaerobic power but also on how efficiently their autonomic system restores physiological balance. The ability to rapidly recover between efforts is influenced by autonomic function, which regulates heart rate recovery and overall cardiovascular fitness [9]. Improved autonomic function can lead to improved heart rate recovery between rounds and ideally improved performance overall. Recent advancements in sports performance technology have enabled the use of sensor-integrated devices to monitor and quantify physiological outputs with high temporal precision [10]. Tools such as cycling ergometers and jump mats now rely on embedded sensors to assess athlete’s performance in real time [11,12]. This growing reliance on sensor-based testing provides researchers and coaches with reliable, noninvasive methods to evaluate combat athletes under controlled conditions.

Short duration, high intensity movements in MMA, such as strikes and takedowns, rely on the ATP-PCr system for immediate energy. However, this system depletes within seconds, requiring efficient recovery and repeated anaerobic output to maintain performance [13]. Understanding the connection between anaerobic energy expenditure, ability to recover, and ability to regenerate power with technical movements performed by MMA fighters would provide paramount understanding into how to train these athletes to optimal potential. Competition-based heart rate, recovery, and other physiological workloads are difficult for training coaches to assess due to the inability for fighters to wear heart rate monitors during fights [14]. Maximal performance tests provide the ability to estimate various energy contributions noninvasively which could be used to adapt athletes’ training pre-competition, pre-fight warm up techniques, mid-fight strategy, and recovery strategies between competitions [13]. These tests have the potential to inform MMA coaches and performance coaches how to optimally prepare their athletes for competition. One study assessed how female and male performance success rates varied based on single high impulse actions versus short duration repeat high impulse actions, determining that female and male amateur MMA fighters have different physiological determinants of performance [15]. These results display differing tactical approaches and training between the two sexes [15]. Sensor-integrated sports science technologies, such as cycling ergometers and contact-based jump systems, allow for noninvasive yet precise performance assessments, making them suitable for MMA-specific testing.

The CMJ is a widely used test for assessing lower body explosive power, a key component of anaerobic performance [16]. The CMJ is a vertical jump test where an athlete starts from a standing position, quickly lowers into a squat, and then jumps as high as possible without stopping. This movement evaluates the stretch-shortening cycle of the lower body muscles, which is essential for explosive power [17]. Lower body power is key in MMA, underpinning essential movements such as striking, takedowns, throws, and explosively standing up from the ground [18,19]. Consistent with this, the medical literature demonstrates that factors including strength, power, and velocity distinguish higher-level MMA competitors from lower-level athletes, highlighting their direct link to performance [18]. Research suggests that CMJ metrics are higher in elite MMA athletes compared to less experienced fighters, displaying the importance of this attribute in the sport [19]. Ideally, MMA training programs should be designed to maximize force production during movement while also minimizing the time required to generate and recover from that force [19]. By improving the timing and control of lower body force, athletes can enhance their striking precision, takedown execution, and ability to rapidly regain position in a fight. Furthermore, CMJ demonstrates the strongest correlation with key markers of explosive power compared to other lower body power tests, making it the most reliable assessment of an athlete’s ability to generate and apply force efficiently [16]. The Just Jump mat, a contact-based system, has been validated for assessing vertical jump performance in comparison to a 3-camera system and is widely used in sports science applications [20,21]. Identifying variations in CMJ amongst weight classes can allow coaches to better tailor training for heavy weight and non-heavy weight fighters. Differences in muscle mass, lean mass, relative strength, and movement efficiency may contribute to variations in jump height and power output, which could impact training across different divisions [22,23,24].

The Wingate Anaerobic Test (WAnT) is a popular assessment for measuring anaerobic power. It is a 30 s all-out bicycle test that assesses peak power, average power, and fatigue index, utilizing the Wingate cycling ergometer, with resistance typically set relative to the participant’s body mass. The Wingate cycling ergometer has been found to be highly reproducible, and a popular tool to assess performance [25]. The Wingate cycle ergometer has been found to be highly reproducible, producing valid and reliable estimates of anaerobic power, making it a suitable ergometer for sport-specific testing [26]. It is currently considered the gold standard for assessing anaerobic capacity, including at the National Hockey League Combine. Previous research highlights the importance of anaerobic power in combat sports. One study using the WAnT found it highly effective in assessing anaerobic capacity, making it a prime tool for evaluating fighters’ endurance, rapid recovery, and sustained power production [27]. However, this study did not specifically include MMA fighters. Another study, investigating anaerobic energy capacity across various sport disciplines, found that anaerobic capacity and power were higher in anaerobic sports like boxing and wrestling compared to predominantly aerobic sports like soccer and long-distance running [28]. When peak power was compared relative to body mass, values were higher in boxers compared to other groups [28]. While these studies do not directly involve MMA athletes, their findings have strong implications for MMA training, particularly among differences across weight classes. However, explicit studies involving MMA athletes remain limited.

Beyond these individual assessments, previous studies have applied a range of performance tests in MMA populations, though the literature remains limited. Previous research demonstrated that countermovement jump variables such as peak power and modified reactive strength index (RSImod) can distinguish higher from lower level competitors, while other work has used incremental load jump squats to evaluate power and velocity characteristics [18,19]. Case studies have also incorporated the WAnT to track training responses in elite fighters [29]. With respect to methodology, force plates are widely considered the gold standard for quantifying CMJ kinetics and kinematics, though jump mats and optical systems have shown strong reliability and are more feasible for field-based testing [30,31,32,33]. Optical systems (e.g., Optojump) and jump mats show excellent reliability and strong validity for CMJ height and flight time, with nearly perfect correlations to force plate data, although raw scores may systematically overestimate values unless corrected [30,31,32,34,35,36]. These field based devices are cost effective, portable, and practical for large scale or in situ athlete monitoring, though they may introduce small systematic biases or reduced accuracy for certain variables compared to force plates [30,31,32,34,35]. Similarly, ergometer-based Wingate testing is well established as a valid and reproducible measure of anaerobic power, including in elite fighters and combat sport athletes. The medical literature demonstrates that the WAnT reliably quantifies peak and mean anaerobic power outputs, with excellent retest reliability and strong criterion validity for tracking training responses and performance adaptations in athletes from combat sports such as boxing, wrestling, judo, and karate and other sports such as ice hockey [28,37]. Collectively, these findings support the application of both laboratory and field based assessments in MMA, while also highlighting the need for further sport specific validation.

The assessment of anaerobic power through both the CMJ and Wingate tests is essential for understanding the physiological demands placed on MMA fighters during competition. Given the sport’s reliance on high intensity, short duration efforts, evaluating anaerobic capacity provides important knowledge about a fighter’s performance potential. While previous studies have investigated anaerobic power in sports like boxing and wrestling, research specific to MMA athletes remains limited. Furthermore, relationships between weight classes, anaerobic power, and recovery dynamics can differ significantly across various MMA athlete profiles. The purpose of this study was to assess peak physiological performance in professional MMA fighters via a Wingate Anaerobic Test (WAnT) and Countermovement Jump (CMJ), and to examine relationships between CMJ (vertical jump) and Wingate performance variables (peak power, average power, fatigue index) in fighters above and below the 83.9 kg (Middleweight 185-pound) weight class [14]. By establishing reliable assessment methods with two sensor-equipped power systems, we can better measure anaerobic cycling and jumping metrics that could enhance individualized training strategies and optimize fight preparation. We hypothesize that significant relationships will exist between CMJ performance and Wingate peak power. Furthermore, we hypothesize that differences in anaerobic power and fatigue indices will be observed between fighters competing above and below the 83.9 kg weight class.

## 2. Materials and Methods

Twelve professional male mixed martial arts fighters (age 29.00 ± 4.80 years, height 180.60 ± 10.10 cm, weight 85.60 ± 13.90 kg) were included in this study. All participants were active, professional mixed martial arts (MMA) athletes at the time of data collection. To be classified as professional, each individual competed in at least one sanctioned professional MMA bout. Additionally, all athletes were engaged in regular, structured training as part of ongoing professional competition schedules and fight preparation. Professional experience ranged from a minimum of 3 years and 5 professional bouts to a maximum of 22 years and 40 professional bouts, reflecting a broad range of career stages across the sample. Height and weight were measured on a standard stadiometer scale. The sample size was based on the number of professional fighters available for testing, and we did not calculate a target sample size in advance using power analysis software. This was a retrospective analysis of deidentified data collected during standard pre-camp performance testing in a controlled laboratory environment. This retrospective study was approved by the University Institutional Review Board. Athletes were grouped into two categories based on competition weight, with lighter fighters weighing less than 83.9 kg and heavier fighters weighing 83.9 kg or more [14]. Each group (light and heavy) consisted of six athletes.

To assess anaerobic performance, two sensor-based systems were used which included the Wattbike Pro and the Just Jump Mat. The Wattbike Pro ergometer (Wattbike Ltd., Nottingham, United Kingdom) uses sensors to measure torque and flywheel revolutions. These signals are used to calculate power output in watts based on torque and angular velocity [26]. For this study, we performed a 30 s anaerobic test on the Wattbike Pro. This protocol is similar to the traditional Wingate Anaerobic Test in that it provides many of the same outcome variables (peak power, mean power, fatigue index), but the two ergometers are not directly interchangeable [26,38]. The Just Jump Mat system (Probotics Inc., Huntsville, AL, USA) is a contact-based testing platform that uses sensors to detect both takeoff and landing [21,35]. The system records flight time and estimates vertical jump height using the equation jump height equals t squared times g divided by eight, where it is flight time in seconds and g is the acceleration due to gravity at 9.81 m per second squared [35].

All testing took place in a single session at the same facility in a laboratory setting between 8AM and 12PM. Prior to testing, all athletes completed standardized familiarization and warm up procedures. For the Wattbike, athletes performed three 6 s all-out sprints to prepare for the 30 s anaerobic test [39]. For the jump mat, athletes completed a dynamic warm-up that included three countermovement jumps before testing [40]. Each athlete completed three countermovement jumps on the Just Jump Mat. Each jump began from a standing position with hands on hips, followed by a rapid downward movement into a squat and immediate upward jump [35]. The jump mat recorded flight time, and the highest jump height from the three trials was used for analysis [35]. Rest periods of at least one minute were provided between jump attempts to minimize fatigue.

Following the jump testing, participants were given a full five minute recovery before they completed a 30 s maximal sprint Wingate Anaerobic Test on the Wattbike Pro [41,42]. Resistance was set based on body weight. Sensor data were used to calculate peak power as the highest five second interval, average power as the mean power over thirty seconds, and a fatigue index as the percentage drop from peak to lowest five second power [38]. Descriptive statistics were used for all subject data. Normality of the dependent variables was assessed using the Shapiro–Wilk test. All variables met the assumption of normal distribution (*p* > 0.05), supporting the use of parametric analyses. Group differences were analyzed using independent samples t-tests, and effect sizes were reported using Cohen’s d. Pearson correlation coefficients were utilized to examine relationships between all CMJ (vertical) and Wingate performance variables (peak power, avg power, fatigue index). Additionally, independent samples *t*-tests were utilized to compare groups (lighter < 83.9 kg; heavier ≥ 83.9) for performance variables and effect sizes were calculated. Power variables were expressed in absolute terms (watts), consistent with previous work that grouped fighters by body mass rather than reporting relative values. All statistical analyses were performed using IBM SPSS Statistics (version 28.0, IBM Corp., Armonk, NY, USA) and statistical significance was set at *p* ≤ 0.05.

## 3. Results

Twelve professional MMA athletes (29.00 ± 4.80 years, 180.60 ± 10.10 cm, 85.60 ± 13.90 kg) demonstrated a strong positive correlation between peak power and fatigue index (r = 0.84, *p* < 0.001), indicating that as peak power increases, fatigue index also tends to increase. Similarly, vertical performance showed a strong positive correlation with avg power (r = 0.71, *p* < 0.001), suggesting that higher countermovement jump heights are associated with higher average power outputs. Vertical also had a moderate positive correlation with peak power (r = 0.61, *p* = 0.004) and fatigue index (r = 0.65, *p* = 0.002), suggesting that greater jump heights are related to higher peak power and fatigue index performance values. Finally, avg power demonstrated a moderate positive correlation with peak power (r = 0.67, *p* = 0.001) and fatigue index (r = 0.46, *p* = 0.045), showing that higher average power is related to higher peak power, but also higher fatigue index values (Table 1).

Descriptive statistics comparing the lighter weight group (<83.9 kg) and the heavier group (≥83.9 kg) demonstrated significant differences for both height and weight (*p* < 0.001) (Table 2). Additionally, performance statistics comparing CMJ and Wingate variables between the lighter (<83.9 kg) and the heavier group (≥83.9 kg) demonstrated a significant difference for peak power (*p* < 0.03) as the heavier group was able to generate a higher performance output. There were trends towards significance for both vertical (*p* = 0.11) and fatigue index (*p* = 0.13), demonstrating a trend towards an advantage for the lighter group when compared to the heavier group; however, this was not significant. No other variables showed significance (*p* > 0.05) (Table 3). Effect sizes for group comparisons ranged from –3.00 (body weight) to +1.00 (vertical). For performance-related variables, effect sizes ranged from –1.52 (peak power) to +1.00 (vertical).

## 4. Discussion

The purpose of this study was to investigate the relationship between vertical jump performance and anaerobic power output in professional mixed martial arts (MMA) athletes, and to explore differences in performance across weight classes. The use of sensor technologies in both the cycling and jump allowed for performance comparison while reinforcing for athlete tracking in applied sport settings. The findings revealed strong positive correlations between countermovement jump (CMJ) height and Wingate anaerobic test (WAnT) performance metrics, including peak and average power [43,44]. This indicates that athletes who demonstrated better explosive power, as measured by the CMJ, also tended to generate greater anaerobic output on the Wingate test. This means that the athletes of the present study who can jump higher generally have better anaerobic performance, which is crucial for explosive movements in a fight like striking or takedowns [2,19,45]. This is consistent with prior work in MMA showing that improvements in CMJ height and power are associated with gains in sprint speed, takedown speed, and other explosive fight-specific tasks [46]. This relationship is not unique to MMA but extends to other combat sports. In wrestling, judo, and Brazilian jiu-jitsu, regression models demonstrate that neuromuscular fitness variables, including vertical jump height and lower-body power, predict up to 73% of variance in Brazilian jiu-jitsu and up to 90% in judo of the variance in high intensity and technical-tactical performance actions during simulated bouts [47]. Similarly, CMJ height has been shown to significantly predict front-kick peak force, explaining about 31% of its variance, and outperforming maximal squat strength as a predictor of kicking performance [48]. In striking sports such as boxing, the association between lower body explosive power and fight specific performance is more nuanced. While CMJ height and lower body strength correlate with punch impact force, studies demonstrate that lower limb power outputs, assessed through CMJ, squat power, and isokinetic strength, are strongly related to punching frequency, velocity, and impact force [45,49,50,51]. Our findings align with these trends, as lighter MMA athletes—who exhibited superior CMJ heights—also demonstrated greater relative anaerobic performance, whereas heavier fighters produced higher absolute Wingate power outputs but at the expense of increased fatigue index. These differences likely reflect the primary mechanical and metabolic demands of each sport: grappling sports emphasize short bursts of maximal lower body force, while striking sports require repeated high velocity actions. By situating MMA athletes within this broader combat sport framework, the strong relationship between CMJ and Wingate observed in our study underscores MMA’s hybrid nature, requiring both the maximal force capacities of grappling sports and the velocity and fatigue resistance of striking sports.

When compared to other combat sports, our MMA sample demonstrated Wingate peak and mean power values lower than those reported in elite national-level Polish boxers, Spanish national Olympic team boxers, and highly trained Chinese boxers [52,53,54]. However, lighter MMA fighters exhibited markedly higher CMJ performance than Spanish national Olympic team boxers and wrestlers, while fatigue index was consistently higher in our cohort [53,55]. Consistent with our MMA data, heavier wrestlers demonstrated greater absolute anaerobic power, whereas lighter athletes showed superior jump performance. Although the wrestling study employed an arm-crank Wingate and our study used a cycle Wingate, the direction of weight-class effects was concordant across sports [55]. Together, these findings suggest that while boxers sustain higher absolute anaerobic outputs with greater efficiency, MMA athletes may sacrifice fatigue resistance for greater explosive capacity. This distinction underscores sport-specific adaptations in how anaerobic power is developed and expressed.

Additionally, the data showed that fighters who produced more peak power during the Wingate test also tended to fatigue more quickly. This suggests that while high power output is important, it may come with a trade-off in the ability to sustain performance. This trade-off has been observed across combat sports including boxing, wrestling, taekwondo, and karate. High-power athletes often show greater decrements during repeated-sprint or jump protocols, reflecting a balance between absolute power capacity and fatigue resistance [27]. Notably, these findings extend across striking (boxing, taekwondo, karate) and grappling (wrestling) disciplines, where intermittent Wingate formats elicit greater peak power through phosphagen contributions, whereas single-bout formats emphasize glycolytic and oxidative metabolism, reinforcing the inherent balance between maximal power and fatigue resistance [27]. A recent resistance-exercise study comparing athletes categorized by their training background (explosive-power vs. maximal-strength) further supports this point. Power athletes produced higher average power and velocity during squat protocols but experienced more rapid fatigue across sets, whereas strength athletes showed greater fatigue resistance despite lower peak outputs [56].

When comparing weight classes, heavier athletes produced significantly more peak power, which aligns with their increased body mass and muscle size. To account for differences in body mass, peak and average power were normalized to body weight (W/kg) and reported as relative peak and average power. No significant differences were observed between weight categories for either metric (*p* ≥ 0.05). This suggests that the higher absolute outputs observed in heavier fighters are primarily attributable to greater body mass rather than superior relative power capacity. In practical terms, lighter athletes appear to generate comparable power when scaled to size, which is consistent with their superior countermovement jump performance. These findings reinforce the importance of reporting both absolute and relative values, as absolute power may drive success in grappling or clinch-heavy exchanges, while relative power is more relevant for repeated explosive actions and speed-based strategies.

Comparable patterns have been observed in Olympic wrestling, where heavy-weight wrestlers demonstrated higher absolute and relative peak power during Wingate testing and greater muscle power outputs than their lighter counterparts, largely explained by increased lean mass and neural activation capacity [55]. Similar results have also been reported in judo athletes, where national and varsity level competitors with greater fat-free mass and muscle mass produced significantly higher peak and mean Wingate power, with strong correlations between fat-free mass and anaerobic power (r = 0.77–0.87) [57]. However, lighter fighters tended to outperform heavier counterparts in CMJ height, consistent with previous findings in wrestlers showing greater relative jump performance in lighter weight categories [55]. This contrast likely reflects underlying biomechanical and physiological factors: heavier athletes benefit from greater absolute muscle mass and cross-sectional area, enabling higher peak power output, whereas lighter athletes possess superior relative strength-to-mass ratios, facilitating greater jump efficiency and recovery capacity. From a training perspective, this suggests that heavier fighters may require conditioning strategies aimed at improving fatigue resistance without compromising absolute force production, while lighter fighters may benefit from programs designed to maximize relative power and sustain repeated explosive efforts.

Our data also suggested that lighter fighters exhibited slightly lower fatigue index scores, implying quicker recovery. Similar trends have been reported in karate and taekwondo, where performance relies heavily on relative lower-limb power, vertical jump capacity, and the ability to sustain repeated high intensity kicking or striking actions, attributes more characteristic of lighter, leaner athletes [58,59]. The relevance of fatigue index in combat sports is reinforced by prior work in karate athletes, where fatigue indices derived from continuous jump testing showed significant correlations with Wingate fatigue index values [60]. Taken together, these findings suggest that weight class differences in MMA reflect broader patterns seen across combat sports: lighter athletes tend to emphasize relative power, speed, and recovery capacity, while heavier athletes excel in absolute force production at the expense of fatigue resistance. These findings support the idea that different weight classes may have distinct physical strengths, and training programs should be adjusted accordingly.

These findings have important implications for performance in MMA, a sport that relies heavily on short bursts of explosive activity for striking, grappling, and takedowns. The observed relationship between vertical jump performance and anaerobic output suggests that CMJ testing could be a simple and accessible tool for coaches to monitor fighters’ explosive power. Additionally, the positive correlation between peak power and fatigue index indicates that athletes who generate high power may gain fatigue more quickly. High output athletes may need targeted conditioning to maintain performance over time. This demonstrates that athletes with high peak power, but also high fatigue index may perform well early in a round but be prone to decline in performance later. Lighter fighters, with better CMJ and lower fatigue, may rely more on speed and quickness which is consistent with their typical fighting styles. Whereas heavier fighters may depend more on power dominant strategies such as fewer but stronger strikes, may benefit from fatigue-focused conditioning to improve sustainability across rounds. Analyses of MMA and boxing reveal that lighter weight classes engage in a higher frequency of distance actions, rapid movement, and striking volume, reflecting a greater reliance on velocity and quickness. Conversely, heavier fighters exhibit more clinch and power-based actions, consistent with their greater absolute strength and muscle mass, but lower relative speed and jump performance [2,61,62]. Interestingly, our cohort produced lower absolute peak and mean Wingate power compared to a world-ranked MMA fighter during a longitudinal fight camp [29]. However, our athletes displayed lower fatigue index values relative to the elite fighter, suggesting that while higher-caliber athletes may achieve greater absolute outputs, this may come at the expense of fatigue resistance [29]. Together, these findings reinforce the Wingate’s utility both for differentiating performance across weight classes and for tracking individual adaptations across training phases. This mirrors the distinction between striking-dominant and grappling-dominant sports: striking sports such as boxing and taekwondo emphasize repeated, high-velocity actions with lower absolute force demands, whereas grappling sports such as wrestling and judo emphasize maximal bouts of force with higher absolute power requirements [55,57,58,61,63]. In this context, MMA athletes must balance both demands, which explains the strong CMJ–Wingate association observed in our study. Additionally, prior work in team-sport athletes has shown that combining CMJ and cycling-based anaerobic tests can effectively track neuromuscular status across training blocks, while frequent CMJ monitoring alone has been used to capture fatigue and adaptation across competitive seasons [64,65,66]. This supports the application of CMJ and Wingate testing together for monitoring training progress in fighters and individualizing conditioning. In practical terms, this suggests that athletes with striking-dominant styles may benefit most from interventions that improve fatigue resistance while maintaining velocity, whereas grappling-dominant athletes may need programs that sustain maximal force production across repeated exchanges. This aligns with recent work proposing comprehensive testing batteries for MMA athletes, which incorporated assessments such as CMJ, Wingate, and strength testing as core measures of performance [6]. For example, an athlete with high CMJ but declining WAnT scores may need more aerobic or anaerobic capacity work, while someone with low CMJ and WAnT may benefit from targeted raw power development.

These findings align with and build upon the existing literature that highlights the anaerobic demands of MMA and related combat sports. For example, the significant relationship observed between CMJ performance and Wingate peak power reinforces previous findings, which noted that elite MMA athletes exhibit higher CMJ metrics, suggesting that lower body power is a key differentiator of performance level [19]. This study further supports this conclusion by demonstrating that CMJ is not only a marker of athleticism, but also predictive of anaerobic capacity as measured by peak power output. Additionally, the observed differences in fatigue index and power output between weight classes add nuance to previous findings, which reported that anaerobic power is significantly higher in combat sport athletes such as boxers and wrestlers compared to those in aerobic sports [28]. The data obtained extend this concept, where heavier fighters may demonstrate greater power but also exhibit higher fatigue index. The observed utility of Wingate testing in evaluating fighters’ anaerobic performance also echoes previous findings, which emphasized the test’s value in assessing short-term power output and fatigue [27]. Although that study did not involve MMA athletes specifically, the findings support the WAnT’s relevance by demonstrating its sensitivity to weight class differences and its correlation with CMJ. The findings also complement prior research demonstrating that physiological determinants of performance differ between male and female amateur MMA fighters [15]. While our study did not assess sex differences, the variability we observed across weight classes similarly emphasizes the need for individualized conditioning approaches based on an athlete’s performance profile. Furthermore, this study contributes to the growing body of literature recognizing the complex energy system demands of MMA [1,2,3,4,5]. The correlations found between CMJ and Wingate variables highlight the importance of training both explosive power and fatigue resistance which are key elements for maintaining high performance across multiple rounds. These results also further confirm previous research emphasizing the importance of anaerobic thresholds and repeat exertion capacity in MMA, particularly given the sport’s repeated transitions between maximal effort and brief recovery [7,13]. In integrating these findings, this study provides additional support for the role of CMJ and Wingate testing as practical tools in evaluating and individualizing training in MMA athletes, especially in the absence of mid-fight physiological monitoring [14]. Together, this growing evidence base supports a shift toward more nuanced performance profiling across fighter types and weight classes to optimize conditioning and fight readiness.

This study was limited by a relatively small sample size, which was not determined in advance using power analysis software, and this may reduce the generalizability of the findings across the MMA athlete population. Another limitation is that the anaerobic cycling test was performed on a Wattbike Pro rather than the traditional Wingate cycle ergometer; although the protocols are similar and provide comparable outcome variables, the two systems are not directly interchangeable and may yield slightly different absolute values. Additionally, while CMJ and Wingate Anaerobic Test (WAnT) metrics provide valuable insights into lower body power and anaerobic performance, the study did not incorporate direct measures of mid-competition performance such as win–loss records, strike output, or grappling exchanges. Therefore, the relevance of lab-based measures to actual fight outcomes is speculative. Other key performance factors such as technical skill, tactical decision-making, psychological preparedness, fight style, and aerobic capacity were also not assessed but are likely to interact with anaerobic power in complex ways. Finally, the cross-sectional design of the study prevents any conclusions about causality or changes over time. Future research should examine these relationships in larger and more diverse samples, ideally across different training phases or competitive levels. Longitudinal studies assessing changes in CMJ and WAnT metrics over the course of a training camp, and how these relate to fight outcomes, would offer stronger evidence for their predictive value. Incorporating performance markers and integrating physiological, technical, and tactical variables could also provide a more holistic understanding of performance in MMA.

## 5. Conclusions

Overall, the results support the use of CMJ and Wingate testing as valuable tools for assessing anaerobic performance in MMA athletes. These assessments can help inform individualized training strategies, particularly when accounting for weight-class-specific strengths and weaknesses. The use of embedded sensor technologies in both Wattbike and Just Jump Mat provided precise, noninvasive data collection methods that are highly applicable to performance monitoring in elite combat sport athletes. Integrating these sensor-based tools into routine athlete profiling may enhance fight preparation strategies and provide ongoing feedback throughout training cycles.

## Figures and Tables

**Table 1:** Correlations of Power Metrics in Professional MMA.

Variable	Vertical (cm)	Peak Power (W)	Avg Power (W)	Fatigue Index (%)
Vertical (cm)	-	0.004 *	<0.001 *	0.002 *
Peak Power (W)	0.004 *	-	<0.001 *	<0.001 *
Avg Power (W)	<0.001 *	<0.001 *	-	0.045 *
Fatigue Index (%)	0.002 *	<0.001 *	0.045 *	-

* Significance set at *p* ≤ 0.05.

**Table 2:** Descriptive Statistics Between Lighter and Heavier Group.

Dependent Variable	Lighter Group (<83.9 kg)	Heavier Group (≥83.9 kg)	T-Stat	*p*-Value	Cohen’s D
Age (years)	29.00 ± 4.80	31.00 ± 6.10	−0.87	0.39	−0.36
Height (cm)	175.20 ± 8.00	188.70 ± 7.10	−4.01	<0.001 *	−1.79
Weight (kg)	76.30 ± 8.70	99.50 ± 6.00	−7.09	<0.001 *	−3.00

* Significance set at *p* ≤ 0.05.

**Table 3:** Performance Results Between Lighter and Heavier Groups.

Dependent Variable	Lighter Group (<83.9 kg)	Heavier Group (≥83.9 kg)	T-Stat	*p*-Value	Cohen’s D
Vertical (cm)	68.15 ± 7.26	59.69 ± 9.46	1.74	0.11	1.00
Peak Power (W)	648.67 ± 106.86	823.17 ± 122.45	−2.63	0.03 *	−1.52
Avg Power (W)	533.50 ± 63.50	552.17 ± 89.20	−0.42	0.69	−0.24
Fatigue Index (%)	29.17 ± 6.18	35.17 ± 6.55	−1.63	0.13	−0.94
Relative Peak Power (W/kg)	8.15 ± 1.59	8.90 ± 1.42	−0.76	0.468	−0.50
Relative Avg Power (W/kg)	6.69 ± 0.95	5.93 ± 1.33	1.06	0.320	0.66

* Significance set at *p* ≤ 0.05.

## Data Availability

Data are not publicly available.

## Abbreviations

The following abbreviations are used in this manuscript:

MMA	Mixed Martial Arts
WAnT	Wingate Anaerobic Test
CMJ	Countermovement Jump
RSImod	modified reactive strength index
